# Coronary Flow Evaluation in Heart Transplant Patients Compared to Healthy Controls Documents the Superiority of Coronary Flow Velocity Reserve Companion as Diagnostic and Prognostic Tool

**DOI:** 10.3389/fcvm.2022.887370

**Published:** 2022-06-24

**Authors:** Annagrazia Cecere, Peter L. M. Kerkhof, Giovanni Civieri, Annalisa Angelini, Antonio Gambino, Angela Fraiese, Tomaso Bottio, Elena Osto, Giulia Famoso, Marny Fedrigo, Enrico Giacomin, Giuseppe Toscano, Roberta Montisci, Sabino Iliceto, Gino Gerosa, Francesco Tona

**Affiliations:** ^1^Department of Cardiac, Thoracic and Vascular Sciences, University of Padua, Padua, Italy; ^2^Department of Radiology and Nuclear Medicine, Amsterdam University Medical Centers, Amsterdam, Netherlands; ^3^Cardiovascular Pathology Unit, Department of Cardiac, Thoracic and Vascular Sciences, University of Padua, Padua, Italy; ^4^Division of Cardiac Surgery, University of Padua, Padua, Italy; ^5^Cardiology, University Heart Center, University Hospital of Zürich, Zurich, Switzerland; ^6^Institute of Clinical Chemistry, University of Zurich, University Hospital of Zürich, Zurich, Switzerland; ^7^Clinical Cardiology, AOU Cagliari, Department of Medical Science and Public Health, University of Cagliari, Cagliari, Italy

**Keywords:** coronary flow reserve, microcirculation, heart transplant, companion metric, prognosis

## Abstract

**Background:**

Distinct contributions by functional or structural alterations of coronary microcirculation in heart transplantation (HT) and their prognostic role have not been fully elucidated. We aimed to identify the mechanisms of coronary microvascular dysfunction (CMD) in HT and their prognostic implications.

**Methods:**

134 patients, surviving at least 5 years after HT, without evidence of angiographic vasculopathy or symptoms/signs of rejection were included. 50 healthy volunteers served as controls. All underwent the assessment of rest and hyperemic coronary diastolic peak flow velocity (DPV_r_ and DPV_h_) and coronary flow velocity reserve (CFVR) and its inherent companion that is based on the adjusted quadratic mean: CCFVR = √{(DPV_r_)^2^ + (DPV_h_)^2^}. Additionally, basal and hyperemic coronary microvascular resistance (BMR and HMR) were estimated.

**Results:**

Based on CFVR and DPV_h_, HT patients can be assigned to four endotypes: endotype 1, discordant with preserved CFVR (3.1 ± 0.4); endotype 2, concordant with preserved CFVR (3.4 ± 0.5); endotype 3, concordant with impaired CFVR (1.8 ± 0.3) and endotype 4, discordant with impaired CFVR (2.0 ± 0.2). Intriguingly, endotype 1 showed lower DPV_r_ (*p* < 0.0001) and lower DPV_h_ (*p* < 0.0001) than controls with lower CFVR (*p* < 0.0001) and lower CCFVR (*p* < 0.0001) than controls. Moreover, both BMR and HMR were higher in endotype 1 than in controls (*p* = 0.001 and *p* < 0.0001, respectively), suggesting structural microvascular remodeling. Conversely, endotype 2 was comparable to controls. A 13/32 (41%) patients in endotype 1 died in a follow up of 28 years and mortality rate was comparable to endotype 3 (14/31, 45%). However, CCFVR was < 80 cm/s in all 13 deaths of endotype 1 (characterized by preserved CFVR). At multivariable analysis, CMD, DPVh < 75 cm/s and CCFVR < 80 cm/s were independent predictors of mortality. The inclusion of CCFVR < 80 cm/s to models with clinical indicators of mortality better predicted survival, compared to only adding CMD or DPV_h_ < 75 cm/s (*p* < 0.0001 and *p* = 0.03, respectively).

**Conclusion:**

A normal CFVR could hide detection of microvasculopathy with high flow resistance and low flow velocities at rest. This microvasculopathy seems to be secondary to factors unrelated to HT (less rejections and more often diabetes). The combined use of CFVR and CCFVR provides more complete clinical and prognostic information on coronary microvasculopathy in HT.

## Introduction

Cardiac allograft vasculopathy (CAV) represents the major determinant of mortality in heart transplantation (HT) patients. This pathologic process is responsible for up to 15% of death annually within the first year following HT, with a cumulative incidence of 50% at 10 years follow-up (1). CAV is characterized by a diffuse luminal narrowing of the graft coronary vasculature secondary to marked concentric intimal hyperplasia and inadequate compensation by outward remodeling (2). The remodeling process involves not only epicardial vessels but also intramural coronaries (3). Microvascular involvement in CAV is widely reported and associated to a poor prognosis (4, 5). Moreover, coronary microvasculopathy may be present in patients with normal epicardial coronary tree (6, 7).

In the past years, CAV diagnosis was based on invasive methods, presenting significant limitations. Coronary angiography, a luminography, demonstrated to underestimate incidence and severity of coronary disease (8), but above all it is unable to recognize the involvement of the coronary microcirculation. Intracoronary Doppler flow wire assessment of coronary flow reserve provided a functional evaluation of microcirculation in CAV patients and it is a reliable marker for major adverse cardiac events (MACEs) (9). However, all these diagnostic tools are invasive, expensive and time-consuming.

The possibility to early detect the pathological changes in microvascular function with non-invasive measurements in HT patients became a clinical priority. In the last years, non-invasive evaluation of microvascular function has been documented (10, 11). Cardiac magnetic resonance imaging (MRI) and stress perfusion MRI allowed the quantification of the myocardial perfusion reserve index (12). Encouraging results come from single-photon emission computed tomography (SPECT) and positron emission tomography (PET) evaluating the distribution of the radionuclide to the various regions of myocardium and the absolute myocardial blood flow, respectively (13, 14). However, these promising methods provided few prognostic implications in HT patients.

We identified a new non-invasive technique based on transthoracic Doppler echocardiography for assessing coronary flow velocity reserve (CFVR) of the left anterior descending coronary artery (LAD) in HT patients (15, 16). CFVR is a crucial functional parameter, that could estimate the physiologic impact of allograft disease on the coronary circulation. CFVR demonstrated to be related to angiographically detectable coronary artery lesion severity and intracoronary Doppler flow wire measurements in ischemic heart disease (17). CFVR proved to be a consistent non-invasive marker able to identify CAV-related cardiac events and a low value was related to a worse prognosis in HT patients (5, 18).

Coronary flow velocity reserve, introduced by Gould in 1974 (19, 20), describes the ability of coronary flow to increase in order to match myocardial metabolic requirements. CFVR is defined as the ratio between coronary flow during hyperemic conditions and coronary flow at rest. This ratio may increase up to 5-times the resting values during exercise and even more by administration of vasodilators (20). Impaired CFVR has been demonstrated in HT patients without epicardial coronary disease (18), suggesting the existence of microvascular structural and/or functional impairment.

Indeed, when epicardial arteries are normal, an impaired CFVR indicates coronary microvascular dysfunction (CMD) which may result from two main mechanisms. Either in the presence of (1) increased baseline coronary flow and concomitant reduced coronary microvascular resistance at rest, or when (2) there is a reduced hyperemic coronary flow due to high microvascular resistance under maximal hyperemia, attributable to impaired vasodilatory function of the coronary microcirculation (21).

The traditional metric CFVR is a dimensionless ratio. However, such ratios are not ideal to explore differences between groups, as same direction changes in numerator and denominator may readily cancel out. Thus, isolated use of CFVR provides incomplete information. Fortunately, this problem can be solved using the data already available. It has been documented that a mathematically derived companion (denoted as CCFVR) complements CFVR (22–24). This strategy was specifically described in patients with psoriasis-related CMD, where adoption of CCFVR enabled a more personal characterization, superior to that achieved by exclusive consideration of CFVR (25).

There are, to our knowledge, no studies investigating the hemodynamic mechanisms underlying the impairment of CFVR in HT patients. Therefore, we aim to elucidate which of the two proposed pathophysiologic mechanisms of CMD is contributing to the impairment of CFVR in HT patients. We also investigate the possible prognostic implications by applying a comprehensive analysis in a comparative study between various microcirculatory patterns and healthy volunteers and evaluate the clinical relevance of the newly introduced CCFVR in these patients.

## Patients and Methods

### Study Population

In this single center cross-sectional study, we selected 134 HT patients who survived at least 5 years after transplantation, having normal biventricular systolic function and without evidence of angiographic CAV or symptoms/signs of rejection. Patients underwent transthoracic Doppler echocardiography to assess CFVR and, within 48 h, coronary angiography to exclude CAV. According to the follow-up protocol of our center, coronary angiography was performed every 2 years. Outcome data were retrieved from medical records. The immunosuppression protocol has been previously described (26, 27). The median time from HT to CFVR determination was 7.1 years (5 to 9.3 years). The non-randomized control group consisted of 50 normal volunteers recruited from institutional personnel who were matched for age and sex. The control subjects were non-invasively studied to determine CFVR and CCFVR, but did not undergo any cardiovascular conditioning program. All control subjects were asymptomatic with no history of heart disease. Exclusion criteria for all subjects included any of the following conditions: cerebral vascular disease, carotid artery bruit, peripheral bruit or abnormal pulse, history of angina or myocardial infarction, alcohol intake > 10 oz per week. All participants had normal ECG at rest and during adenosine-induced hyperemia.

The study protocol was approved by the institutional ethical committee. All participants gave written informed consent.

### Acute Rejection Scores

Acute graft rejection was monitored by periodical endomyocardial biopsies, after standardized protocols (weekly during the first month, biweekly until the third month, monthly until the first year; when in the presence of grade 2 rejection, then in the following 10–15 days) (5). After first year of HT, endomyocardial biopsies were performed only in the presence of clinical suspicion of acute rejection. On the basis of modification of the ISHLT grading, a rejection score was assigned for each patient (28). For each patient the following scores were calculated: rejection score (RS) in the total follow-up (TRS); RS in the first year (RS 1st year), RS including only severe grades (≥3A) in the total follow-up (SevTRS); first-year RS including only severe grades (1st yrSevRS). All scores were subsequently normalized for the number of biopsies performed in each patient.

### Angiography/Diagnosis of Cardiac Allograft Vasculopathy

Cardiac catheterization was performed within 48 h of CFVR evaluation. A cardiologist unaware of the clinical and echocardiographic findings reviewed angiograms. As previously reported, a qualitative grading system was utilized: grade I, normal angiogram; grade II, luminal irregularities or diameter reduction < 30%; grade III, diameter reduction < 50%; grade IV, diameter reduction ≥ 50% and/or diffuse narrowing of small vessels (5). The presence of angiographic grade II or greater defined CAV and patients with CAV at baseline were excluded from the study.

### Echocardiography and Coronary Flow Velocity Reserve Assessment

Transthoracic Doppler echocardiography (Vivid 7, GE Medical System, Inc., Horten, Norway) was performed. From two-dimensional guided M-mode echocardiograms, left ventricular (LV) dimensions were measured by American Society of Echocardiography (ASE) convention; LV mass was calculated by the adjusted ASE method (29) and indexed for body surface area or height. Ejection fraction was measured and diastolic dysfunction was defined according to ASE criteria (29). Coronary images were obtained in the distal part of the left anterior descending artery (LAD) with 7-MHz transducer. The approach for the distal part was already validated and consisted first in obtaining a short axis of the left ventricular apex and anterior groove to search for coronary flow. When a diastolic blood flow was recognized, the transducer was rotated clockwise to obtain the best long axis of color flow. Alternatively, a modified 2-chamber view was obtained by sliding the transducer superiorly and medially from an apical 2-chamber position. Then, search for color-coded blood flow was made over the epicardial part of the anterior wall (15). After recordings of peak coronary diastolic flow velocity (DPV) at rest (DPV_r_), adenosine was intravenously infused (140 μg⋅kg^–1^⋅min^–1^) for 3 min, obtaining hyperemic DPV (DPV_h_). CFVR was the ratio of DPV_h_ and DPV_r_. A CFVR ≤ 2.5 was considered abnormal and marker of CMD, and the population was dichotomized according to this cut-off (5, 30). Assessment of CFVR only in one coronary artery (LAD) is a limitation of our study, but this choice was imposed by the non-invasive echocardiographic approach. CFVR measurement by transthoracic echocardiography, indeed, was originally validated with intracoronary Doppler flow wire in the LAD (17) and there is, to our knowledge, no routine echocardiographic approach to assess CFVR in other coronary arteries. As regards heterogeneity of microvascular dysfunction, a modest degree of correlation of CFVR between LAD and right coronary artery/left circumflex artery has been reported in studies using positron emission tomography (31): it is therefore reasonable to assume that CFVR in the LAD reflects global coronary flow reserve. As regards DPV_h_ there is not, to our knowledge, a defined cut-off value in HT patients. We therefore decided to use the median value of DPV_h_ in our study group (75 cm/s) as cut-off. Moreover, we recorded heart rate (HR) at rest and during hyperemia (HR_r_ and HR_h_), systolic and diastolic arterial pressure at rest (SAP_r_ and DAP_r_) and during hyperemia (SAP_h_ and DAP_h_). In order to minimize differences in cardiac work, CFVR was normalized to the corresponding rate-pressure product (rpCFVR) by dividing the CFVR by the rate-pressure product (an index of cardiac work), multiplied by a linear factor of 10.000 in each patient.

The evaluation of coronary flow involved the assessment of microvascular resistance. Coronary microvascular resistance (mmHg⋅s/cm) was obtained from the mean blood pressure measured in the arm by sphygmomanometer (mean pressure = [2 × diastolic + systolic] /3) divided by DPV, both at rest and during hyperemia. In particular, we assessed coronary microvascular resistance in the basal (BMR, basal microvascular resistance) and in the hyperemic condition (HMR, hyperemic microvascular resistance). Finally, the arteriolar resistance index (ARI), defined as the difference between BMR and HMR, was calculated. ARI was considered a marker of vascular compliance and expressed the vessels’ capability to dilate under maximal hyperemia (32).

### The Companion to Coronary Flow Velocity Reserve in the Velocity Domain

**FIGURE 1:**
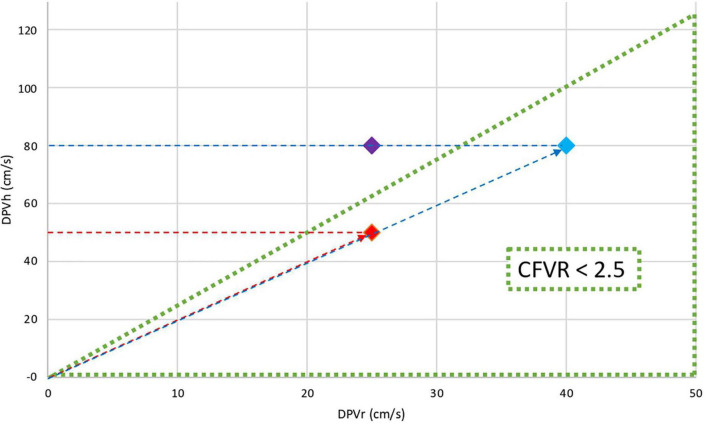
Cartesian and polar coordinates in the flow velocity domain. The triangular area represents coronary microvascular dysfunction (CFVR ≤ 2.5). The graph shows the DPV_h_ (cm/s) versus DPV_r_ (cm/s) for three different patients: Red dot and Blue dot have the same CFVR (2.0) while Purple dot has normal CFVR (3.2). Clearly, the CFVR values for Red and Blue dots are identical, although the flow velocity levels both at rest and during hyperemia are substantially different. This discrepancy indicates that CFVR alone cannot adequately define myocardial perfusion conditions. The difference between various pathophysiological states can be further quantified by calculating their individual distance to the origin, that is, the length of the red dotted line with the arrowhead and the blue dotted line with the arrowhead. This companion, denoted as CCFVR (cm/s), equals the hypotenuse (Red dot CCFVR = 55.9 cm/s; Blue dot: CCFVR = 89.4 cm/s).

While any simple ratio such as CFVR refers to only two variables, it is a convenient approach to depict DPV_h_ versus DPV_r_ in the velocity domain diagram. Then polar coordinates CFVR and CCFVR can be mapped on the familiar Cartesian plane (22, 25) (Figure 1). Any point in polar coordinates is uniquely characterized by these two alternative components: (1) the slope (angle) reflects CFVR; (2) the corresponding distance from the origin to that specific point, here referred to as CCFVR. This distance equals the length of the hypotenuse subtended by the Cartesian data pair (DPV_r_, DPV_h_) as shown in Figure 1.

The numerical value of CCFVR can easily be obtained, being defined as (22):


$$C\,C\,F\,V\,R=\surd \left\{{\left({\text{DPV}}_{\text{r}}\right)}^{2}+{\left({\text{DPV}}_{\text{h}}\right)}^{2}\right\}$$

Apart from a factor 1/√2 = 0.71, this value equals the so-called quadratic mean. Similarly as for DPV_h_, there is no defined cut-off for CCFVR in HT patients and we used the median value of the study group (80 cm/s) as cut-off.

### Clinical Outcomes

Two independent investigators, specifically assigned to this task and blinded to coronary flow assessment, carefully reviewed clinical outcomes. The average follow-up was 15.1 ± 5.8 years from the HT (range 6.2–28.5 years). For this study we considered the cardiovascular mortality as the main clinical outcome. Data about mortality were collected from the medical records. In addition, further information was obtained by evaluating hospital discharge cards and the personal status (i.e., alive/dead) recorded in the medical information system of our region.

### Statistical Analysis

Continuous variables with no/mild skew were presented as mean ± SD; skewed measures were represented as median with first and third quartiles (Q1-Q3). Discrete variables were summarized as frequencies and percentages. The distribution of the data was analyzed with a 1-sample Kolmogorov-Smirnov test. Categorical variables were compared by the χ^2^ test or the Fisher exact test as appropriate. Continuous data were compared by use of the two-tailed unpaired *t* test (for normally distributed data sets) or the Mann-Whitney *U* test (for skewed variables). Univariable Cox regression analysis was performed for all clinical and echocardiographic variables, and the variables with a *p* < 0.10 were included in a multivariable Cox regression analysis to identify independent predictors of the end point: hazard ratio (HR) and 95% confidence intervals were calculated. Kaplan–Meier curves were constructed to estimate the cumulative event-free survival and compared by the log-rank test. To evaluate the incremental value of DPV_h_ and CCFVR on top of clinical and standard echocardiographic parameters, likelihood ratio testing was performed, as well as calculation of the overall C-statistic as proposed by Harrell et al. (33) as an analog of the area under the receiver operating characteristic curve for survival analysis. Furthermore, we assessed the impact of adding DPV_h_ and CCFVR to a basic model using the continuous net reclassification improvement. The intraobserver and interobserver reproducibilities of CFVR were evaluated by linear regression analysis and expressed as correlation coefficients (r), the standard error of estimates (SEE) and the intraclass correlation coefficient (ICC). These reproducibilities were assessed by repeating the CFVR evaluation twice, 1 h apart, by the same operator (G.F.) in all patients and by another operator (F.T.) in all patients, before and after treatment. Reproducibility was considered satisfactory if the intraclass correlation coefficient was between 0.81 and 1.0.

All tests were two-sided and statistical significance was accepted if the null hypothesis could be rejected at *p* < 0.05. Data were analyzed with SPSS software version 24.0 (SPSS, Inc., Chicago, IL, United States).

## Results

### Study Population

The study population was subdivided in four endotypes on the basis of CFVR and DPV_h_. As previously reported, a CFVR ≤ 2.5 was considered abnormal (5, 30); therefore, the study population was dichotomized according to this cut-off in patients with preserved CFVR (CFVR > 2.5) and patients with impaired CFVR (CFVR ≤ 2.5). In the study group the median of DPV_h_ was 75 cm/s; we utilized this cut-off to identify patients with impaired DPV_h_ (DPV_h_ ≤ 75 cm/s) and preserved DPV_h_ (DPV_h_ > 75 cm/s). The median value of CCFVR in the study group was 80 cm/s and we selected it as cut-off to classify patients with impaired CCFVR (CCFVR ≤ 80 cm/s) and preserved CCFVR (CCFVR > 80 cm/s).

Microvascular coronary flow parameters of the control group and HT patients are reported in Table 1. HT patients showed lower DPV_h_ (*p* < 0.0001) than controls, with lower CFVR (*p* < 0.0001), even if normal, and lower CCFVR (*p* < 0.0001). Moreover, HT patients differ significantly from the control group in terms of HMR (*p* = 0.02), ARI (*p* = 0.01), CMD (*p* < 0.0001), DPV_h_ < 75 cm/s (*p* = 0.003) and CCFVR < 80 cm/s (0.007). The difference between DPV_h_ and DPV_r_, defined as ΔDPV, is also significantly different between HT patients and controls, in both absolute (DPV_h_ – DPV_r_) and percentwise relative [(DPV_h_-DPV_r_)⋅100/DPV_r_] terms (both *p* < 0.0001).

**TABLE 1:** Microvascular coronary flow parameters in healthy subjects and heart transplant patients.

	Control group (*n* = 50)	HT patients (*n* = 134)	*p*
DPV_r_, cm/s	26 (19–30)	25 (21–33)	0.77
DPV_h_, cm/s	92 (74–105)	75 (58–89)	<0.0001
CFVR	3.5 (3.0–4.1)	2.9 (2.2–3.4)	<0.0001
BMR, mmHg⋅s/cm	4.17 (3.48–5.15)	4.01 (3.07–5.00)	0.22
HMR, mmHg⋅s/cm	1.13 (0.95–1.40)	1.23 (1.02–1.61)	0.02
ARI, mmHg⋅s/cm	3.00 (2.31–3.95)	2.66 (1.86–3.46)	0.01
rpCFVR	3.1 (2.4–3.7)	3.3 (2.6–4.1)	0.21
Δ DPV, cm/s	64 (52–75)	49 (33–62)	<0.0001
Δ DPV, %	250 (200–306)	199 (116–243)	<0.0001
CCFVR, cm/s	95 (78–110)	80 (63–94)	<0.0001
CMD, n (%)	0 (0)	78 (58.2)	<0.0001
DPV_h_ < 75 cm/s, n (%)	13 (26)	68 (50.7)	0.003
CCFVR < 80 cm/s, n (%)	14 (28)	57 (42)	0.007

*ARI, arteriolar resistance index; BMR, basal microvascular resistance; CCFVR, companion coronary flow velocity reserve; CFVR, coronary flow velocity reserve; CMD, coronary microvascular dysfunction; DPV_h_, hyperemic diastolic peak velocity; DPV_r_, rest diastolic peak velocity; HMR, hyperemic microvascular resistance; rpCFVR, CFVR normalized to the rate-pressure product; Δ DPV, difference between hyperemic and basal diastolic peak velocities.*

### Hemodynamic and Microvascular Coronary Flow Parameters in the Study Population

Based on CFVR and DPV_h_, the study population was subdivided in four different endotypes: endotype 1 (n: 32 pts), preserved CFVR and impaired DPV_h_; endotype 2 (n: 60 pts), both preserved CFVR and DPV_h_; endotype 3 (n: 31 pts), both impaired CFVR and DPV_h_; endotype 4 (n: 11 pts), impaired CFVR and preserved DPV_h_. These endotypes revealed four different microvascular responses to heart transplantation, as summarized in Table 2.

**TABLE 2:** Hemodynamic and microvascular coronary flow parameters in heart transplant patients stratified by 4 endotypes.

	Preserved CFVR		Impaired CFVR		*p* for differences		
			
	Impaired DPV_h_ (endotype 1) *n* = 32	Preserved DPV_h_ (endotype 2) *n* = 60	Impaired DPV_h_ (endotype 3) *n* = 31	Preserved DPV_h_ (endotype 4) *n* = 11	endotype 1 vs. endotype 2	endotype 1 vs. endotype 3	endotype 3 vs. endotype 4
DPV_r_, cm/s	20 (19–23)	26 (23–30)	25 (22–34)	41 (39–48)	<0.0001	<0.0001	<0.0001
DPV_h_, cm/s	62 (55–69)	89 (81–100)	45 (40–63)	86 (76–94)	<0.0001	<0.0001	<0.0001
CFVR	3.0 (2.7–3.4)	3.3 (2.9–3.7)	1.9 (1.6–2.0)	2.1 (1.9–2.2)	0.008	<0.0001	0.08
BMR, mmHg⋅s/cm	5.15 (4.49–5.8)	3.72 (3.1–4.54)	3.91 (3–4.88)	2.3 (2.04–2.89)	<0.0001	<0.0001	<0.0001
HMR, mmHg⋅s/cm	1.51 (1.29–1.7)	1.03 (0.85–1.12)	1.83 (1.49–2.2)	1.14 (1.01–1.23)	<0.0001	0.004	<0.0001
ARI, mmHg⋅s/cm	3.5 (3.13–4.21)	2.65 (2.14–3.35)	1.85 (1.4–2.89)	1.36 (0.99–1.52)	<0.0001	<0.0001	0.01
rpCFVR	3.6 (3–4.2)	3.9 (3.2–4.5)	2.1 (1.6–2.5)	2.2 (2.1–2.5)	0.14	<0.0001	0.35
Δ DPV, cm/s	42 (36–48)	63 (56–73)	21 (13–31)	46 (36–52)	<0.0001	<0.0001	<0.0001
Δ DPV, %	200 (170–243)	235 (198–274)	91 (48–105)	105 (85–120)	0.005	<0.0001	0.11
CCFVR	66 (57–72)	92 (84–103)	54 (47–68)	94 (87–105)	<0.0001	0.005	<0.0001

*ARI, arteriolar resistance index; BMR, basal microvascular resistance; CCFVR, companion coronary flow velocity reserve; CFVR, coronary flow velocity reserve; CMD, coronary microvascular dysfunction; DPV_h_, hyperemic diastolic peak velocity; DPV_r_, rest diastolic peak velocity; HMR, hyperemic microvascular resistance; rpCFVR, CFVR normalized to the rate-pressure product; Δ DPV, difference between hyperemic and basal diastolic peak velocities.*

**FIGURE 2:**
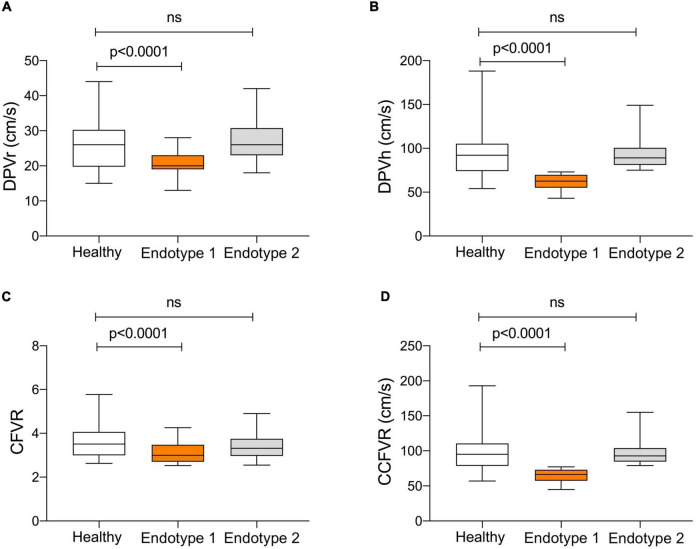
Comparison of microvascular pattern in HT patients with preserved CFVR (endotype 1 and endotype 2) and control group. Endotype 2 and controls share a comparable microvascular pattern, while endotype 1 has significantly lower DPV_r_ (Panel **A**; *p* < 0.0001), DPV_h_ (Panel **B**; *p* < 0.0001), CFVR (Panel **C**; *p* < 0.0001) and CCFVR (Panel **D**; *p* < 0.0001). This figure highlights how, even among patients with preserved CFVR, there are substantial differences in coronary microvascular flow parameters.

Endotype 1 is characterized by preserved CFVR and impaired DPVh, demonstrating the presence of low rest flow that significantly increases during hyperemia. The low rest flow is due to a high BMR that decreases during hyperemia, allowing to achieve a normal CFVR. Conversely, endotype 2 had both preserved CFVR and DPV_h_ with a good vascular response to hyperemia. In comparison to endotype 1, endotype 2 presented a higher rest and hyperemic flow (*p* < 0.0001) with lower microvasculature resistance (*p* < 0.0001). In presence of a preserved CFVR, as observed in these two endotypes, the main pathophysiological difference is due to a high BMR and HMR that produce, in endotype 1, a low rest flow and low DPV_h_ as well. There aren’t significant differences in cardiac work between the two endotypes in terms of rpCFVR (*p* = 0.14). Although with a normal CFVR, endotype 1 doesn’t present a similar hemodynamic and microvascular flow pattern when compared to control group, as summarized in Figure 2. On the contrary, there are no significant differences in terms of DPV_r_, DPV_h_, CFVR and CCFVR between endotype 2 and control group, thus sharing a comparable microvascular flow.

The microvascular flow differences between the various endotypes reflect distinct clinical characteristics, as summarized in Table 3. In fact, endotype 1 has more frequently ischemic heart disease pre-HT (40.6% vs. 25%; *p* = 0.04), diabetes (25% vs. 10%; *p* = 0.05), older age at HT (55 ± 11 vs. 47 ± 15; *p* = 0.01), higher donor age (37 ± 13 vs. 30 ± 12; *p* = 0.02) and severe TRS (0.23 ± 0.08 vs. 0.59 ± 0.09; *p* = 0.01) than endotype 2.

**TABLE 3:** Clinical characteristics in different endotypes.

	Endotype 1 (*n* = 32)	Endotype 2 (*n* = 60)	Endotype 3 (*n* = 31)	Endotype 4 (*n* = 11)	p 1 vs. 2	p 1 vs. 3
Deaths, n (%)	13 (40.6)	16 (26.7)	14 (45.2)	6 (54.5)	0.03	0.7
Time from HT, years	6 ± 0.9	7.5 ± 0.7	8.6 ± 1.1	8.3 ± 1.0	0.2	0.08
Recipient male, n (%)	28 (87.5)	40 (66.7)	27 (87.1)	10 (90.9)	0.03	0.9
Donor male, n (%)	24 (75)	28 (46.7)	19 (61.3)	8 (72.3)	0.009	0.2
Sex mismatch, n (%)	6 (18.8)	24 (40)	8 (25.8)	2 (18.2)	0.03	0.5
IHD pre-HT	13 (40.6)	15 (25)	12 (38.7)	6 (54.5)	0.04	0.8
Diabetes, n (%)	8 (25)	6 (10)	9 (29)	1 (8.2)	0.05	0.6
Age at HT, years	55 ± 11	47 ± 15	52 ± 14	49 ± 14	0.01	0.3
Donor age, years	37 ± 13	30 ± 12	35 ± 14	29 ± 12	0.02	0.5
SevTRS	0.23 ± 0.08	0.59 ± 0.09	0.40 ± 0.07	0.56 ± 0.17	0.01	0.04

*HT, heart transplantation; IHD, ischemic heart disease; SevTRS, severe total rejection score.*

Patients with low rest and low hyperemic flow belonged to endotype 3, presenting both impaired CFVR and DPV_h_. Similarly to endotype 1 also regarding to the clinical characteristics (Table 3), endotype 3 had low DPV_r_ but also presented a low hyperemic flow due to a higher HMR (*p* = 0.004) (Table 2). So, endotype 3 included patients without any vasodilatory capability in the hyperemia conditions, due to high vascular resistance. Finally, patients with impaired CFVR but preserved DPV_h_ composed endotype 4. In comparison to endotype 3, these patients presented high rest and hyperemic flow (*p* < 0.0001) with lower vascular resistance (*p* < 0.0001), in absence of significant differences in cardiac work (*p* = 0.35) (Table 2). This condition could be related to the early exhaustion of coronary dilatation reserve, avoiding a further vasodilatation in the just amplified microvasculature. Similarly to endotype 3, these patients failed to vasodilate because they had a large rest dilation in the presence of low resistances.

### Correlations Between Coronary Flow and Long-Term Cardiovascular Free Survival

**FIGURE 3:**
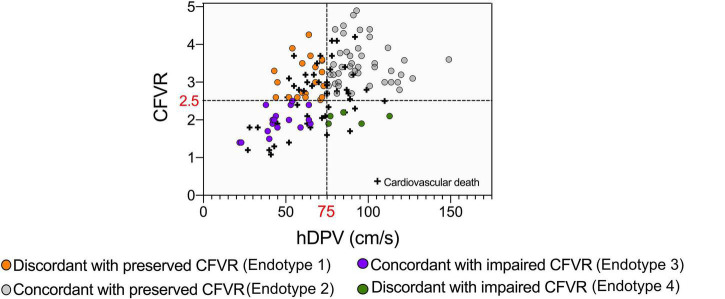
Cardiovascular mortality in HT patients subdivided on the basis of CFVR and DPV_h_. Cardiovascular mortality observed in endotype and endotype 2 shows, as expected, the lowest level (27%). Endotype 1 and 3, even if with different CFVR patterns, have comparable mortality rates (41 and 45%, respectively), while endotype 4 has the overall highest mortality (55%).

**FIGURE 4:**
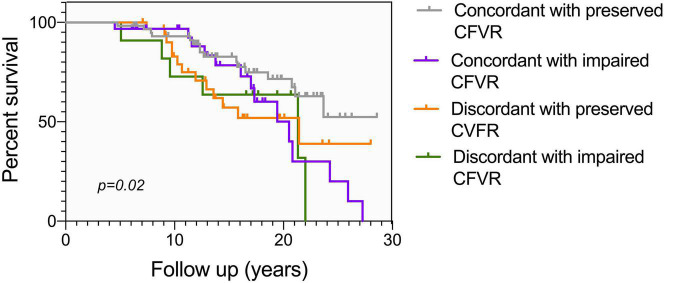
Kaplan Meier mortality curve according to different CFVR endotypes, conditional at 5-years survival. As in Figure 3, endotype 2 has the lowest mortality, while endotype 1 and endotype 3 share a comparable survival. Endotype 4 has the highest mortality, with no patients surviving more than 21 years after HT. Different endotypes can significantly predict survival (*p* = 0.02).

Differences between survivors and non-survivors are presented in Table 4. Male sex (*p* = 0.04), previous ischemic heart disease (*p* = 0.05), onset of any-grade CAV (*p* = 0.02) and degree of severe rejection within the first year (*p* = 0.03) are clinical characteristics of the recipients, which factors are associated with mortality. As regards microvascular coronary flow parameters, lower DPV_h_ (*p* = 0.02), higher HMR (*p* = 0.01), lower CFVR (*p* = 0.007) and lower CCFVR (*p* = 0.03) are more frequent among non-survivors. The prevalence of CMD is not significantly different among survivors and non-survivors (*p* = 0.1), but, interestingly, non-survivors have a significantly higher prevalence of impaired CCFVR (*p* = 0.02). As shown in Table 3 and Figures 3, 4, we observed that cardiovascular mortality was noted in every endotype, even in the presence of preserved CFVR. Concordant patients with preserved CFVR (endotype 2) presented a lower rate mortality when compared with discordant ones of endotype 1 (27% vs. 41%; *p* = 0.03). Nevertheless, as a dimensionless ratio-based metric, CFVR is not always expected to correctly stratify the risk of cardiovascular mortality in HT patients with different microvascular patterns. In fact, mortality rate in endotype 1 (discordant with preserved CFVR) is quite similar to endotype 3 (concordant with impaired CFVR) (41% vs. 45%; *p* = 0.7). Moreover, while patients in both endotype 3 and 4 have impaired CFVR, those in endotype 4 had a higher mortality rate (55% vs. 45%, *p* = 0.03) (Table 3 and Figure 4).

**TABLE 4:** Clinical, hemodynamic and microvascular coronary flow parameters in survivors and non-survivors heart transplant patients.

	Overall (*n* = 134)	Survivors (*n* = 85)	Non-survivors (*n* = 49)	*p*
**Clinical recipient characteristics**				
Age at transplant, years	50 ± 14	49 ± 14	53 ± 13	0.14
Male, n (%)	105 (78.3)	62 (72.9)	43 (87.8)	0.04
Time post-transplant, years	7.5 ± 0.8	6.9 ± 0.6	8.4 ± 0.8	0.13
Body mass index, Kg/m^2^	25.6 ± 3	25.6 ± 4	25.5 ± 3	0.89
Hypertension, n (%)	98 (73.1)	61 (71.7)	37 (75.5)	0.65
Obesity, n (%)	18 (13.4)	12 (14.1)	6 (12.2)	0.77
Diabetes, n (%)	21 (15.6)	15 (17.6)	6 (12.2)	0.41
Dyslipidemia, n (%)	68 (50.7)	43 (50.5)	25 (51)	0.89
IHD pre-transplant, n (%)	46 (34.3)	24 (28.2)	22 (44.9)	0.05
Renal failure, n (%)	113 (84.3)	77 (90.5)	36 (73.4)	0.49
CAV onset, n (%)	52 (38.8)	15 (30.6)	37 (43.5)	0.13
CAV grade (0,1,2,3), %	61.2, 26.1,7.5,5.2	69.4,12.2,12.2,6.1	56.5,34.1,4.7,4.7	0.02
LVEF, %	64 ± 6	63 ± 5	64 ± 7	0.74
TRS	1.141 ± 0.5	1.061 ± 0.5	1.261 ± 0.4	0.24
RS 1st year	1.098 ± 0.5	1.028 ± 0.5	1.215 ± 0.6	0.07
Sev TRS	0.454 ± 0.3	0.411 ± 0.3	0.505 ± 0.4	0.37
Sev RS 1st year	0.454 ± 0.4	0.387 ± 0.4	0.570 ± 0.5	0.03
**Clinical donor characteristics**				
Donor age, years	33 ± 13	32 ± 12	34 ± 14	0.44
Male, n (%)	79 (58.9)	53 (62.4)	26 (53.1)	0.29
Sex mismatch, n (%)	40 (29.8)	21 (24.7)	19 (38.8)	0.08
Ischemic time, minutes	179 ± 55	178 ± 54	182 ± 56	0.65
Body mass index, Kg/m^2^	24.3 ± 3.0	24.6 ± 2.8	23.7 ± 3.5	0.26
**Medication use, n (%)**				
Aspirin	70 (52.2)	42 (49.4)	28 (57)	0.51
Beta-blockers	56 (41.7)	37 (43.5)	19 (38.7)	0.47
ACEi or ARB	58 (43.2)	38 (44.7)	20 (40.8)	0.62
Diuretics	36 (26.8)	21 (24.7)	15 (30.6)	0.35
Statins	102 (76.1)	68 (80)	34 (69.3)	0.10
Calcineurin inhibitor	113 (84.3)	72 (84.7)	41 (83.6)	0.89
mTOR inhibitor	50 (37.3)	34 (40)	16 (32)	0.41
**Microvascular coronary flow parameters**				
DPV_r_, cm/s	25 (21–33)	25 (21–30)	25 (21–35)	0.55
DPV_h_, cm/s	75 (59–89)	79 (61–94)	71 (56–81)	0.02
CFVR	2.9 (2.2–3.4)	3 (2.4–3.5)	2.7 (1.9–3.1)	0.007
BMR, mmHg.s/cm	4.01 (3.07–5.0)	4.02 (3.1–4.96)	3.85 (3.02–5.09)	0.75
HMR, mmHg.s/cm	1.23 1.03–1.61)	1.12 (0.94–1.59)	1.38 (1.11–1.66)	0.01
ARI, mmHg.s/cm	2.66 (1.86–3.45	2.73 (1.95–3.47)	2.61 (1.55–3.46)	0.25
rpCFVR	3.3 (2.6–4.1)	3.3 (2.7–4.2)	3.0 (2.3–3.9)	0.07
Δ DPV, cm/s	49 (33–62)	53 (34–66)	43 (32–53)	0.003
Δ DPV, %	192 (116–243)	202 (146–250)	175 (95–212)	0.01
CCFVR, cm/s	80 (64–94)	84 (67–97)	74 (61–87)	0.03
CMD, n (%)	56 (41.7)	31 (36.5)	25 (51)	0.1
DPV_h_ < 75 cm/s, n (%)	68 (50.7)	36 (42.3)	32 (65.3)	0.01
CCFVR < 80 cm/s, n (%)	67 (50)	36 (42.3)	31 (63.3)	0.02
Endotype (1,2,3,4),%	23.9,44.8,23.1,8.2	22.4,51.7,20,5.9	26.5,32.7,28.6,12.2	0.04
Discordant endotypes, n (%)	40 (30)	17 (20)	23 (47)	0.001

*ACEi, ACE-inhibitors; ARB, angiotensin receptor blockers; ARI, arteriolar resistance index; BMR, basal microvascular resistance; CAV, coronary allograft vasculopathy; CCFVR, companion coronary flow velocity reserve; CFVR, coronary flow velocity reserve; CMD, coronary microvascular dysfunction; DPV_h_, hyperemic diastolic peak velocity; DPV_r_, rest diastolic peak velocity; Δ DPV, difference between hyperemic and basal diastolic peak velocities; HMR, hyperemic microvascular resistance; HT, heart transplantation; IHD, ischemic heart disease; RS, rejection score; rpCFVR, CFVR normalized to the rate-pressure product; SevTRS, severe total rejection score; TRS, total rejection score.*

**FIGURE 5:**
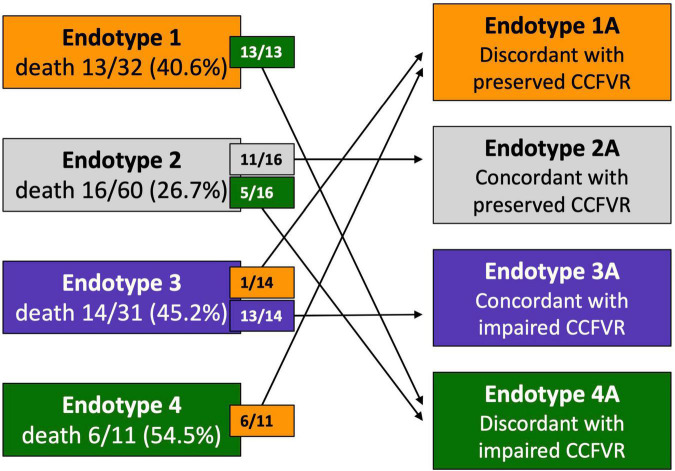
Reclassification of HT patients on the basis of CCFVR which allows to further stratify mortality risk among HT patients. For example, endotype 1 has preserved CFVR but its mortality risk is comparable to endotype 3 (with impaired CFVR), making CFVR an incomplete predictor of mortality. If we reclassify patients using CCFVR, all the deaths of endotype 1 (preserved CFVR) are shifted to endotype 4A (preserved CFVR but impaired CCFVR). Interestingly, also 5 deaths from endotype 2 (also preserved CFVR) are assigned to endotype 4A using CCFVR.

Considering that CFVR and DPV_h_ cannot always correctly stratify the risk of cardiovascular mortality, patients were reclassified in 4 variant endotypes on the basis of previously defined cut-offs, i.e., CCFVR (preserved: > 80 cm/s) and CFVR (preserved: > 2.5), yielding: endotype 1A, preserved CCFVR and impaired CFVR; endotype 2A, preserved CCFVR and CFVR; endotype 3A, impaired CCFVR and CFVR; endotype 4A, impaired CCFVR and preserved CFVR. The proposed application of CCFVR permitted a redistribution of cardiovascular death cases. In particular, CCFVR totally reassigned cardiovascular deaths of endotype 1 to endotype 4A (preserved CFVR with impaired CCFVR), underlining the incremental value of the newly introduced companion. Indeed, CCFVR is able to recognize patients at increased risk, even when presenting with preserved CFVR. Also five cases of death for endotype 2 were reassigned to endotype 4A when using CCFVR (Figure 5).

**FIGURE 6:**
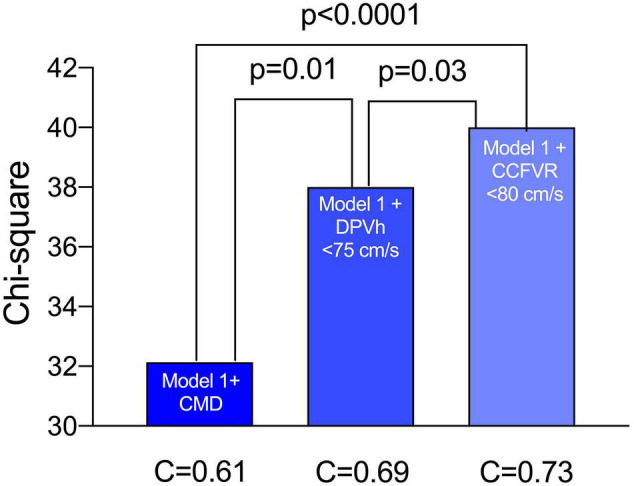
Performance of various survival prediction models among HT patients. Age at transplant, time post-transplant, onset of CAV, donor age and ischemic time are independent predictors of survival (referred to as Model 1). We evaluated the incremental prognostic value of CMD (defined as CFVR < 2.5), DPV_h_ < 75 cm/s and CCFVR < 80 cm/s to Model 1 and found that CCFVR is significantly more accurate than CMD (*p* < 0.0001) and DPV_h_ (*p* = 0.03). The inclusion of CCFVR to clinical predictors of mortality permitted therefore better prediction of survival among HT patients.

At multivariable survival analysis, age at transplant (*p* = 0.003), post-transplant period (*p* < 0.0001), onset of CAV (*p* = 0.006), and ischemic time (*p* = 0.01) were found to be independent clinical predictors of mortality. As regards microvascular coronary flow parameters, CFVR (*p* = 0.03), presence of CMD (*p* = 0.04), DPV_h_ < 75 cm/s (*p* = 0.001) and CCFVR < 80 cm/s (*p* = 0.003) were also independent predictors of mortality in HT patients (Table 5). Consequently, we evaluated the impact of strategies including CMD, DPVh < 75 cm/s and CCFVR < 80 cm/s to a prognostic model covering only the independent clinical predictors of mortality (referred as Model 1). The inclusion of CCFVR < 80 cm/s to Model 1 permitted better prediction of survival in HT patients, compared to only adding CMD (*p* < 0.0001) or DPVh < 75 cm/s (*p* = 0.03) (Figure 6).

**TABLE 5:** Univariate and multivariable Cox proportional hazard regression analysis to identify independent predictors of cardiovascular mortality.

	Univariable HR (95% CI)	*P-value*	Multivariable HR (95% CI)	*P-value*
**Clinical recipient characteristics**				
Age at transplant	1.04 (1.02–1.07)	<0.0001	1.04 (1.01–1.08)	0.003
Male	2.20 (1.06–5.20)	0.06		
Time post-transplant	1.16 (1.08–1.24)	<0.0001	1.19 (1.10–1.29)	<0.0001
Body mass index	1.07 (0.99–1.17)	0.08		
Hypertension	1.71 (1.25–3.71)	0.16		
Obesity	1.88 (0.76–4.64)	0.16		
Diabetes	1.08 (0.45–2.57)	0.86		
Dyslipidemia	1.31 (1.42–2.44)	0.39		
IHD pre-transplant	2.13 (1.20–3.83)	0.009	1.39 (1.31–2.55)	0.27
Renal failure	1.41 (0.53–3.73)	0.48		
CAV onset	2.17 (1.15–4.09)	0.01	2.60 (1.32–5.11)	0.006
CAV grade	0.82 (0.57–1.18)	0.30		
LVEF	0.98 (0.92–1.04)	0.64		
TRS	1.42 (0.51–3.94)	0.50		
RS 1st year	1.27 (0.81–2.01)	0.29		
Sev TRS	1.30 (0.44–3.87)	0.63		
Sev RS 1st year	1.12 (0.67–1.87)	0.66		
**Clinical donor characteristics**				
Donor age	1.04 (1.02–1.07)	<0.0001	1.02 (0.99–1.05)	0.09
Male	0.82 (0.46–1.44)	0.49		
Sex mismatch	1.51 (0.85–2.69)	0.15		
Ischemic time	1.008 (1.003–1.01)	0.003	1.007 (1.002–1.013)	0.01
Body mass index	1.01 (0.88–1.15)	0.89		
**Medication use**				
Aspirin	0.89 (0.84–1.01)	0.81		
Beta-blockers	0.91 (0.88–1.02)	0.62		
ACEi or ARB	0.99 (0.97–1.02)	0.58		
Diuretics	1.001 (0.98–1.003)	0.41		
Statins	0.81 (0.74–0.93)	0.12		
Calcineurin inhibitor	1.002 (1.001–1.004)	0.79		
mTOR inhibitor	0.91 (0.88–0.97)	0.39		
**Microvascular coronary flow parameters**				
DPV_r_	1.02 (0.98–1.05)	0.30		
DPV_h_	1.01 (1.00–1.02)	0.05		
CFVR	1.45 (1.06–2.00)	0.01	1.54 (1.02–2.32)	0.03
BMR	0.94 (0.74–1.19)	0.64		
HMR	1.18 (0.81–1.71)	0.37		
ARI	0.88 (0.69–1.13)	0.32		
rpCFVR	1.07 (0.87–1.33)	0.15		
Δ DPV, cm/s	1.01 (1.00–1.03)	0.01	1.02 (1.005–1.04)	0.01
Δ DPV, %	1.004 (1.00–1.007)	0.02	0.99 (0.95–1.03)	0.88
CCFVR	1.01 (1.002–1.02)	0.09		
CMD*	1.88 (1.06–3.31)	0.02	1.51 (1.22–2.80)	0.04
DPV_h_ < 75 cm/s*	2.48 (1.37–4.50)	0.003	7.35 (2.29–23.80)	0.001
CCFVR < 80 cm/s*	2.62 (1.41–4.71)	0.002	6.83 (1.95–23.93)	0.003
Coronary flow pattern		0.02		0.29
Endotype 1	2.31 (1.34–2.98)		1.15 (1.08–2.34)	
Endotype 3	2.30 (1.11–5.90)		2.05 (1.73–2.46)	
Endotype 4	1.12 (1.08–2.36)		1.06 (1.01–1.84)	
Discordant endotypes	2.71 (1.54–4.78)	0.001	2.50 (1.39–4.50)	0.002

*Multivariable analysis performed separately with CMD, DPV_h_ < 75 cm/s or CCFVR < 80 cm/s. ACEi, ACE-inhibitors; ARB, angiotensin receptor blockers; ARI, arteriolar resistance index; BMR, basal microvascular resistance; CAV, coronary allograft vasculopathy; CCFVR, companion coronary flow velocity reserve; CFVR, coronary flow velocity reserve; CMD, coronary microvascular dysfunction; DPV_h_, hyperemic diastolic peak velocity; DPV_r_, rest diastolic peak velocity; Δ DPV, difference between hyperemic and basal diastolic peak velocities; HMR, hyperemic microvascular resistance; HT, heart transplantation; IHD, ischemic heart disease; RS, rejection score; rpCFVR, CFVR normalized to the rate-pressure product; SevTRS, severe total rejection score; TRS, total rejection score.

### Intraobserver and Interobserver Reproducibilities of Coronary Flow Velocity Reserve

The intraobserver reproducibility was high (*r* = 0.93, SEE = 0.10); the mean difference was −0.005; the upper and lower limits of agreement between the measurements were + 0.16 (95% CI, +0.09 to + 0.21) and −0.18 (95% CI, −0.24 to −0.13), respectively; and the ICC was 0.974. The interobserver reproducibility was also high (*r* = 0.90, SEE = 0.11); the mean difference was −0.016, the upper and lower limits of agreement between the two measurements were + 0.31 (95% CI, +0.23 to + 0.43) and −0.33 (95% CI, −0.41 to −0.21), respectively, and the ICC was 0.963.

## Discussion

The present study revealed that HT patients with normal systolic function and no evidence of CAV presented coronary microvascular impairment. Microvascular impairment, mainly related to reduced CFVR, demonstrated to be related to new onset of CAV and cardiovascular deaths (18, 34). The possibility to non-invasively identify markers of microvascular dysfunction, responsible for cardiovascular mortality in HT patients, is challenging for clinicians.

We aimed to identify mechanisms of coronary microvascular impairments in HT patients and their potential prognostic implications by applying a comprehensive and multiparametric analysis.

The combination of CFVR and DPV_h_ permitted to identify four patterns of coronary microvascular impairment, each representing distinct adaptive mechanisms concerning microvascular hemodynamics after HT. The observed variety of these patterns of coronary microvascular impairment could have potential prognostic implications.

First of all, impairment of CFVR could be attributed to two situations: presence of epicardial coronary stenosis, or pure microvascular dysfunction in the absence of coronary artery disease. In contrast to other studies, we included in the present study only patients without CAV (18, 35). For this reason, in the absence of coronary artery stenosis, impairment of CFVR reflects a reduced coronary reserve with a loss of the vasodilatory ability of microvasculature (36). This impairment of CFVR in HT patients is related to a worse prognosis. The pathophysiological mechanisms have not been fully elucidated (37), but could be attributable to structural and/or functional remodeling. The first condition, realized by concordant endotype 3, is related to a reduced resting and hyperemic flow velocity, due to high resistance during rest and hyperemia. This was already reported in invasive studies regarding patients with CMD and non-obstructive coronary artery disease, where lower DPV_r_ was found to be associated with lower DPV_h_ and with a more adverse myocardial function, possibly through a mechanism of chronic myocardial tissue hypoperfusion (38). In our hypothesis, the high resting and hyperemic resistances may refer to a possible structural remodeling of microvasculature, related to progressive vessel fibrosis, responsible for the consequent reduced flow. The fibrotic replacement of coronary microvasculature limits adenosine-induced vasodilation. In this situation, CFVR and DPV_h_ may be both reduced due to structural alterations of microvasculature. Further studies with histological assessment of microvessels in endomyocardial biopsies will help to corroborate this hypothesis.

Interesting is the pathway of discordant endotype 4, which is characterized by an impaired CFVR, but preserved DPV_h_. In contrast to endotype 3, this condition is related to a functional remodeling of coronary microvasculature. In fact, in the presence of increased resting and hyperemic coronary flow and decreased resistance, impaired CFVR is the result of a permanent vasodilation of coronary microvasculature. Loss of capability to further vasodilate in presence of adenosine is related to a myocardial high-stress condition, producing a massive reduction of peripheral resistance. This phenomenon could be explained by an adaptive response to high basal myocardial oxygen requirement, producing a massive recruitment of coronary reserve, also in resting condition. This compensative response seems to be shared with Cushing syndrome, characterized by cortisol excess and systemic arterial stiffness, underlining the presence of a common adaptive response to myocardial stress (39). This functional remodeling with the loss of vasodilatory capacity and resulting permanent high coronary flow could lead, time by time, to a structural microvascular impairment, responsible for a coronary flow decrease and onset of symptomatic heart failure (40). For this reason, the microvascular pattern of impaired CFVR represents two faces of the same coin. In fact, the clinical result is the impaired CFVR, related in the first case to a fibrotic vessel replacement and in the second case to a constant requirement of high flow, in order to supply to the excessive myocardium oxygen requirement. Time by time, the functional remodeling could be irreversible, leading to a fibrotic replacement of coronary microvasculature remodeling (41).

If it’s quite reasonable to assume that reduced CFVR is linked to an impairment of the coronary microcirculation, then the condition of preserved CFVR deserves careful consideration.

The state of preserved CFVR includes the situation of reduced DPV_h_ (discordant endotype 1) and normal DPV_h_ (concordant endotype 2). The concordant endotype 2 represents patients with the better adaptive pattern and the consequently lowest rate of mortality, because of its both preserved CFRV and DPV_h_. Conversely, discordant endotype 1 presented reduced DPV_h_ for the high resting resistance that significantly reduces during adenosine infusion, permitting to reach a normal CFVR. Nevertheless, both the conditions of preserved CFVR cannot be assimilated to a physiological state. In fact, the comparison of microvascular and hemodynamic pattern of both endotypes with preserved CFRV (endotype 1 and endotype 2) with control group revealed significant differences. In particular, endotype 1 tends to present lower resting and hyperemic coronary flow with lower CFVR, even if normal, and lower CCFVR than controls. Also, in presence of normal CFVR, patients who belong to endotype 1 tend to have more frequently diabetes, pre-HT ischemic heart disease, older age at HT and higher donor age than endotype 2. As a common cardiovascular risk, male sex (both recipient and donor) represents a clinical determinant for adverse microvascular response in endotype 1. These different clinically relevant factors suggest that the observed microvascular outcomes in the endotype 1 may reflect not only the adaptive response to HT, but also the individual characteristics of patients predisposing to a high resistance (42). The presence of two different microvascular patterns in presence of preserved CFVR underlines that CFVR isn’t capable to accurately discriminate a good microvascular response. Indeed, the identification of different endotypes even among patients with preserved CFVR allows a better risk stratification of these patients.

Confirming that preserved CFVR isn’t synonymous for normal coronary flow pattern, we noticed that mortality rate in endotype 1 (with preserved CFVR) is quite similar to mortality reached in the endotype 3 (with impaired CFVR). Again, the identification of different endotypes allows to identify those patients at higher risk. Due to this result, it’s reasonable to assume that CFVR alone may be inadequate to really identify HT patients with a greater mortality risk and this highlights the need for new indexes of microvascular function. Interestingly, the use of CCFVR, a newly introduced parameter with physical dimensions (cm/s) based on the quadratic mean of CFVR, allowed a redistribution of cardiovascular deaths in each endotype. In particular, the rate of cardiovascular death reached in the endotype 1 is totally reassigned to endotype 4A (preserved CFVR but impaired CCFVR), again suggesting that CFVR isn’t able to correctly recognize patients with an impairment of coronary microvasculature. On the contrary, the use of its related companion CCFVR is able to identify HT patients with greater mortality risk, not captured by CFVR. This happens because CFVR, being a ratio of velocities, is dimensionless and, therefore, not always able to capture structural CMD with low flow velocities and high resistances. Indeed, changes in the numerator and denominator occurring in the same direction may cancel each other out (23). A companion metric (CCFVR) may be better suited to identify differences at similar CFVR values, as already proved in psoriasis patients (25). As a consequence of a deeper patient characterization, CCFVR is also a stronger predictor of mortality compared to CFVR and DPV_h_ and it allows a more accurate risk stratification of HT patients, just by applying available data without the need to perform additional measurements.

On the basis of these results, a non-invasive multiparametric approach is more comprehensive when CCFVR is included, and advocated to better evaluate coronary microcirculation in HT patients.

## Conclusion

Coronary microvascular impairment is related to CAV and cardiovascular deaths in HT patients. The possibility to non-invasively detect microvascular changes, in order to predict prognosis in HT patients, represents a major challenge for clinicians. Even if CFVR is documented to be a non-invasive marker able to identify patients with poor coronary reserve, we demonstrated its inadequacy to correctly identify patients at greater risk of cardiovascular deaths. A normal CFVR isn’t a warranty of good functionality of coronary microvasculature and it could hide detection of microvascular damage, as shown by the finding of different microvascular flow parameters in different endotypes. In presence of preserved CFVR, the microvasculopathy, identified with high flow resistance and low flow velocities at rest, seems to be secondary to factors unrelated to HT (i.e., less rejections and more often diabetes). Being a dimensionless ratio, CFVR may miss predicting some cases of early death, yet captured by CCFVR. Thus, the combined use of CFVR and CCFVR could provide more complete clinical information on coronary microvasculopathy and guide HT patient management.

## Data Availability Statement

The raw data supporting the conclusions of this article will be made available by the authors, without undue reservation.

## Ethics Statement

The studies involving human participants were reviewed and approved by the University of Padua. The patients/participants provided their written informed consent to participate in this study.

## Author Contributions

AC and PK generated figures and tables, and drafted the manuscript. GC assisted with generation of figures and editing of the manuscript. SI, GG, and FT contributed to the conception and design of the research as well as editing of the manuscript. AA, GT, and MF contributed to the conception and design of the project. EO contributed to the conception and design of the project, and editing of the manuscript. GF, TB, and AG contributed to the conception and design of the research, generation of figures and tables, and drafting to editing of the manuscript. AF, GF, and EG critically revising the manuscript and designed the study. All authors contributed to the article and approved the submitted version.

## Conflict of Interest

The authors declare that the research was conducted in the absence of any commercial or financial relationships that could be construed as a potential conflict of interest.

## Publisher’s Note

All claims expressed in this article are solely those of the authors and do not necessarily represent those of their affiliated organizations, or those of the publisher, the editors and the reviewers. Any product that may be evaluated in this article, or claim that may be made by its manufacturer, is not guaranteed or endorsed by the publisher.
